# Hyperglycemia-induced STING signaling activation leads to aortic endothelial injury in diabetes

**DOI:** 10.1186/s12964-023-01393-w

**Published:** 2023-12-21

**Authors:** Ying An, Kang Geng, Hong-ya Wang, Sheng-rong Wan, Xiu-mei Ma, Yang Long, Yong Xu, Zong-zhe Jiang

**Affiliations:** 1 Department of Endocrinology and Metabolism, The Affiliated Hospital of Southwest Medical University, Luzhou, Sichuan PR China 646000; 2 Metabolic Vascular Disease Key Laboratory of Sichuan Province, Luzhou, 646000 Sichuan China; 3 Sichuan Clinical Research Center for Nephropathy, Luzhou, 646000 Sichuan China; 4 Department of Plastic and Burn Surgery, The Affiliated Hospital of Southwest Medical University, Luzhou, Sichuan PR China 646000; 5 Academician (Expert) Workstation of Sichuan Province, The Affiliated Hospital of Southwest Medical University, Luzhou, Sichuan PR China 646000

**Keywords:** Hyperglycemia, STING, Aortic endothelial cells, Diabetes

## Abstract

Hyperglycaemia-induced endothelial dysfunction is a key factor in the pathogenesis of diabetic microangiopathy and macroangiopathy. STING, which is a newly discovered regulator of innate immunity, has also been reported to play an important role in various metabolic diseases. However, the role of STING in diabetes-induced endothelial cell dysfunction is unknown. In this study, we established a diabetic macroangiopathy mouse model by streptozotocin (STZ) injection combined with high-fat diet (HFD) feeding and a glucotoxicity cell model in high glucose (HG)-treated rat aortic endothelial cells (RAECs). We found that STING expression was specifically increased in the endothelial cells of diabetic arteries, as well as in HG-treated RAECs. Moreover, genetic deletion of STING significantly ameliorated diabetes-induced endothelial cell dysfunction and apoptosis in vivo. Likewise, STING inhibition by C-176 reversed HG-induced migration dysfunction and apoptosis in RAECs, whereas STING activation by DMXAA resulted in migration dysfunction and apoptosis. Mechanistically, hyperglycaemia-induced oxidative stress promoted endothelial mitochondrial dysfunction and mtDNA release, which subsequently activated the cGAS-STING system and the cGAS-STING-dependent IRF3/NF-kB pathway, ultimately resulting in inflammation and apoptosis. In conclusion, our study identified a novel role of STING in diabetes-induced aortic endothelial cell injury and suggested that STING inhibition was a potential new therapeutic strategy for the treatment of diabetic macroangiopathy.

Video Abstract

**Supplementary Information:**

The online version contains supplementary material available at 10.1186/s12964-023-01393-w.

## Introduction

Diabetes has become one of the most serious and common chronic diseases today. According to the latest IDF data, there were more than 500 million people with diabetes in the world in 2021, which means that more than 10.5% of adults worldwide are suffering from this disease [1]. Cardiovascular problems account for the majority of morbidity and mortality in diabetic patients, and up to 80% of diabetic patient deaths are directly related to vascular illnesses [2].

Chronic hyperglycaemia, which is one of the key pathogenic factors in diabetes, plays an important role in the development of diabetic vascular complications, which pose a serious threat to the lives of diabetic patients [3]. Endothelial cell dysfunction, which is characterized by impaired endothelium-dependent vasodilatation, chronic inflammation, leukocyte adhesion, hyperpermeability and cellular senescence, is the initial stage and an important prognostic indicator of diabetic vascular complications [4, 5]. Many previous reports have revealed the mechanisms by which hyperglycaemia induces endothelial dysfunction through inflammatory or different cell death pathways [6]. One of the most important mechanisms is hyperglycaemia-induced oxidative stress, which could be mediated by various pathways, such as the activation of protein kinase C, an increase in polyol flux and the hexosamine pathway, leading to the disruption of vascular homeostasis [7]. As one of the major producers of reactive oxygen species (ROS), mitochondria can also be injured by the overproduction of mtROS under hyperglycaemic conditions, such as a decrease in membrane potential and impaired integrity of the mitochondrial membrane [8]. It has been reported that compromised mitochondrial integrity leads to the leakage of mitochondrial DNA (mtDNA) into the cytosol and activates the stimulator of interferon genes (STING) pathway, resulting in pathological inflammation in chronic kidney disease (CKD) [9]. However, the role of hyperglycaemia-induced mtDNA release in diabetic vascular endothelial cell injury is unknown.

As a newly discovered DNA sensor-related protein, STING is activated following DNA binding with cGAS. Many studies have revealed the roles of the cGAS-STING system in regulating autoimmune diseases such as rheumatoid arthritis, Aicardi Goutières syndrome and systemic lupus erythematosus [10, 11] and other inflammatory diseases [12]. Recently, a growing number of studies have shown that the cGAS-STING signalling pathway is abnormally activated by metabolic dysfunction-induced cytosolic mtDNA release during metabolic diseases. In a mouse model of nonalcoholic fatty liver disease (NAFLD), STING knockout reduced mtDNA-induced NF-κB activation and inflammatory factor expression in hepatic Kupffer cells [13]. In *db/db* mice, which is a classic T2DM mouse model, hyperlipidaemia-induced mtDNA release activates the cGAS-STING system in cardiomyocytes, which leads to heart injury through IRF3/NF-kB signalling [14]. In addition, recent studies have shown a link between STING signalling and vascular inflammation. DNA from damaged aortic smooth muscle cells (SMCs) is engulfed by macrophages, and the cytosolic DNA activates STING and its downstream target IRF3, leading to the development of sporadic aortic aneurysm and dissection (AAD) [15]. During atherogenesis, single-stranded DNA (ssDNA) accumulation in macrophages accelerates vascular inflammation and macrophage activation through the induction of inflammatory molecule expression [16]. In lung endothelial cells, lipopolysaccharide (LPS)-induced GSDMD cleavage promotes mtDNA release into the cytoplasm, thus activating the cGAS-STING pathway and ultimately inhibiting endothelial cell proliferation [17]. In diet-induced obesity, the STING-IRF3 signalling pathway is activated in mouse adipose tissue, which leads to endothelial inflammation and macrophage infiltration in adipose tissue [18]. However, the role of STING signalling in diabetic vascular disease is still unclear.

Given that hyperglycaemia-induced mitochondrial dysfunction can lead to mtDNA release and that the cGAS-STING system can be activated by cytosolic mtDNA, it is important to identify whether hyperglycaemia contributes to aortic endothelial cell injury through mtDNA-induced cGAS-STING activation. In the present study, we established a diabetic mouse model by HFD feeding and STZ injection. Interestingly, we found that STING expression was specifically increased in the aortic endothelial cells of diabetic mice. STING inhibition ameliorated diabetes-induced endothelial cell injury in vivo and high glucose-induced endothelial cell dysfunction in vitro. Further study revealed that high glucose-induced cytosolic mtDNA release activated cGAS-STING signalling and the downstream inflammatory pathway in aortic endothelial cells. Our novel observations suggest that hyperglycaemia-induced STING activation contributes to aortic endothelial cell injury in diabetes, indicating that functional inhibition of STING may be a therapeutic strategy for diabetic vascular disease.

## Materials and methods

### Animals

Wild-type C57BL/6 mice (WT) and STING-knockout mice (C57BL/6 N-Tmem173^em1cyagen^; STING^−/−^ mice) were purchased from Cyagen (Suzhou, Jiangsu). Sixty percent of the high-fat feed was purchased from Trophic, Nantong. A high-fat diet combined with streptozotocin (STZ, Solarbio Science & Technology Co., Beijing, China) was used to establish a type 2 diabetes model by intraperitoneal injection of 50 mg/kg STZ for 5 consecutive days. All animal experiments were performed under the following conditions: room temperature of 23 ± 1 °C, relative humidity of 60% ± 10%, and alternating 12-hour light-dark cycles in individually ventilated cages. The animal experiments were approved by the Institutional Animal Ethics Committee of Southwest Medical University (No. 20220225–014). The experiments were performed according to the National Institutes of Health (NIH) Guide for the Care and Use of Laboratory Animals.

### Cell culture and treatments

Rat aortic endothelial cells (RAECs) (ATCC, USA) were cultured in low-sugar DMEM (HyClone, Logan, Utah, USA) containing 10% foetal bovine serum (FBS, ScienCell, USA) and 1% penicillin/streptomycin (PS, Beyotime, Shanghai, China) at 37 °C and 5% CO_2_. Human aortic endothelial cells (HAECs) (iCell, Shanghai) were cultured in ECM (ScienCell, USA) medium at 37 °C and 5% CO_2_. High glucose (40 mmol/L), DMXAA (50 μg/ml) and C176 (5 nmol/L) stimulation for 72 h was performed, and the effects on high glucose-induced RAECs were studied.

### Isolation of mouse aortic ECs

Mouse aortic endothelial cells were isolated from male WT and STING^−/−^ mice (3 months old) that were perfused with PBS containing 1000 U/mL heparin through the left ventricle after being anesthetized. The aorta was removed and placed in a culture dish filled with ice-cold PBS to remove the surrounding adipose tissue. The aorta was cut into 8–10 small segments, placed in a collagenase IA (Sigma, USA) solution at 37 °C and gently shaken for 20 minutes. After the digestion was complete, ECM (ScienCell, USA) medium was added to terminate the digestion. The separated cells were centrifuged at 800×g for 10 minutes and resuspend in ECM medium. The cells were placed in a 37 °C incubator with 5% CO_2_ for 1 hour. The cells were washed with PBS to remove impurities and nonadherent cells. Then, new ECM medium was added, the cells were further cultured. This study used cells from the 1st to 3rd generations.

### Histological assay

Briefly, the tissue was fixed in 4% paraformaldehyde and then paraffin embedded. The aortic tissue was cut into thin slices with a thickness of 4 μm and incubated overnight at 37 °C. The sections were then dewaxed and rehydrated. The structure of the aortic tissue was then observed using H&E staining.

### Immunohistochemical staining

The experiments were performed as previously described [14]. Paraffin sections were dewaxed, hydrated and stained with primary antibodies against VEGF (1:100, Beyotime), IL-18 (1:100, Beyotime) and IL-1β (1:100, Beyotime). The sections were stained with biotin-labelled goat anti-rabbit IgG or biotin-labelled anti-mouse IgG and then treated with horseradish enzyme-labelled ovalbumin from Streptomyces (Beijing ZSGB Biotechnology Co., Ltd. China).

### Immunofluorescence staining

Aortic tissue/RAECs were incubated overnight at 4 °C with antibodies against mitofilin (1:100, Abcam, UK), dsDNA (1:100, Santa Cruz, USA), STING (1:100, CST, USA), IRF3 (1:100, Santa Cruz, USA), p65 (1:100, Santa Cruz, USA), and CD31 (1:100, Abcam, UK). Then, the cells were incubated with FITC/Cy3 fluorescent dye-conjugated secondary antibodies for 1 h in the dark. DAPI (Abcam, UK) was used for nuclear staining. A confocal microscope (Leica, Germany) was used for observation.

### qRT–PCR analysis

Total RNA was extracted from aortic tissue and RAECs using TRIzol reagent (Invitrogen, USA). Reverse transcription was performed using ReverTra Ace qPCR RT Master Mix (fq − 201, TOYOBO), and qRT–PCR was performed using a QuantiInova SYBR Green PCR Kit (Qiagen, Germany). qRT–PCR was performed using the Analytikjena qTOWER 3 g Real-Time PCR System (Jena, Germany) according to the manufacturer’s instructions. GAPDH was used as an internal reference gene to normalize target gene expression, and the data shown are representative of three independent experiments. Relative expression to the internal reference gene was calculated using the 2^-ΔΔCt^ method.

### Mitochondrial DNA (mtDNA) isolation and transfection

mtDNA was isolated from RAECs using the Mitochondrial DNA Isolation Kit (Abcam, UK). Briefly, 5 × 10^6^ cells were collected, washed with ice-cold PBS and lysed on ice in Cytosol Extraction Buffer for 10 minutes. The cells were homogenized using an ice-cold dounce tissue grinder and centrifuged at 700 x g for 10 minutes at 4 °C to remove nuclear granules and intact cells. The supernatant was centrifuged at 10,000 x g for 30 minutes, the supernatant was removed, and the pellets were resuspended in Cytosol Extraction Buffer and centrifuged at 10,000 x g for 30 minutes. The pellet (mitochondria) was resuspended and lysed in mitochondrial lysis buffer for 10 minutes. The proteins were digested with a mixture of enzymes at 50 °C for at least 2 hours. The mtDNA was precipitated with anhydrous ethanol and stored at − 20 °C. mtDNA (3 μg/well) was transferred to a small dish with a diameter of 6 cm and incubated with Lipofectamine 3000 (Thermo Fisher, US) for 24 hours.

### ELISA and DNA isolation

cGAMP concentration in RAECs and mouse aorta was determined by cGAMP ELISA KIT (Huadeyibo, China) according to the manufacturer’s instructions. Whole cell genomic DNA was extracted by centrifugal column using DNA extraction kit (FOREGENE, China).

### Migration assay

Cell migration was detected by scratch assays. When the RAECs in the 6-well plate reached 100% confluence, the cells were scratched with the tip of a 1 ml pipette needle. After 48 h, the area of the scratch was imaged using ImageJ software and measured.

### ROS, JC-1 and TUNEL assays

The method was as described in our previous study [19]. ROS levels in RAECs were measured by a DCFH-DA fluorescent probe according to the ROS assay kit protocol (Beyotime, China). TUNEL staining of paraffin sections was performed according to the TUNEL kit protocol (Beyotime, China). JC-1 staining of RAECs was performed according to the JC-1 assay kit protocol (Solarbio, China).

### Western blot analysis

For western blotting, aortic tissue and RAECs proteins were extracted using extraction buffer (RIPA). Protein samples were separated by sodium dodecyl sulfate–polyacrylamide gel electrophoresis (SDS–PAGE) and transferred onto PVDF membranes (Millipore, USA). The membranes were incubated with 5% BSA to block other contaminants, incubated with primary antibodies at 4 °C overnight, washed three times with TBST and incubated with secondary antibodies for 1 h at room temperature. Finally, the protein bands were visualized using ECL luminescent reagent. Cytoplasmic proteins were normalized to tubulin. The following primary antibodies were used: cGAS (1:1000, Santa Cruz, USA), STING (1:1000, CST, USA), p65 (1:3000, CST, USA), p-p65 (1:1000, CST, USA), IRF3 (1:3000, CST, USA), p-IRF3 (1:1000, CST, USA), IL-1β (1:1000, CST, USA), CD31 (1:1000, Beyotime, China), VEGF (1:1000, Beyotime, China), IL-18 (1:1000, Beyotime, China), IL-1β (1:1000, Beyotime, China), and tubulin (1:500, CST, USA).

### Statistical analysis

The data are expressed as the means ± standard deviations (SD). Comparisons between two groups were performed using student’s test. Statistical analyses were evaluated using GraphPad Prism 9 (GraphPad Software, USA). A value of *p* < 0.05 was considered statistically significant (*P < 0.05, **0.001 < *P* < 0.01, and ***P < 0.001).

## Results

### cGAS-STING is upregulated in the aortic endothelial cells of diabetic mice and high glucose-treated RAECs

**Fig. 1:**
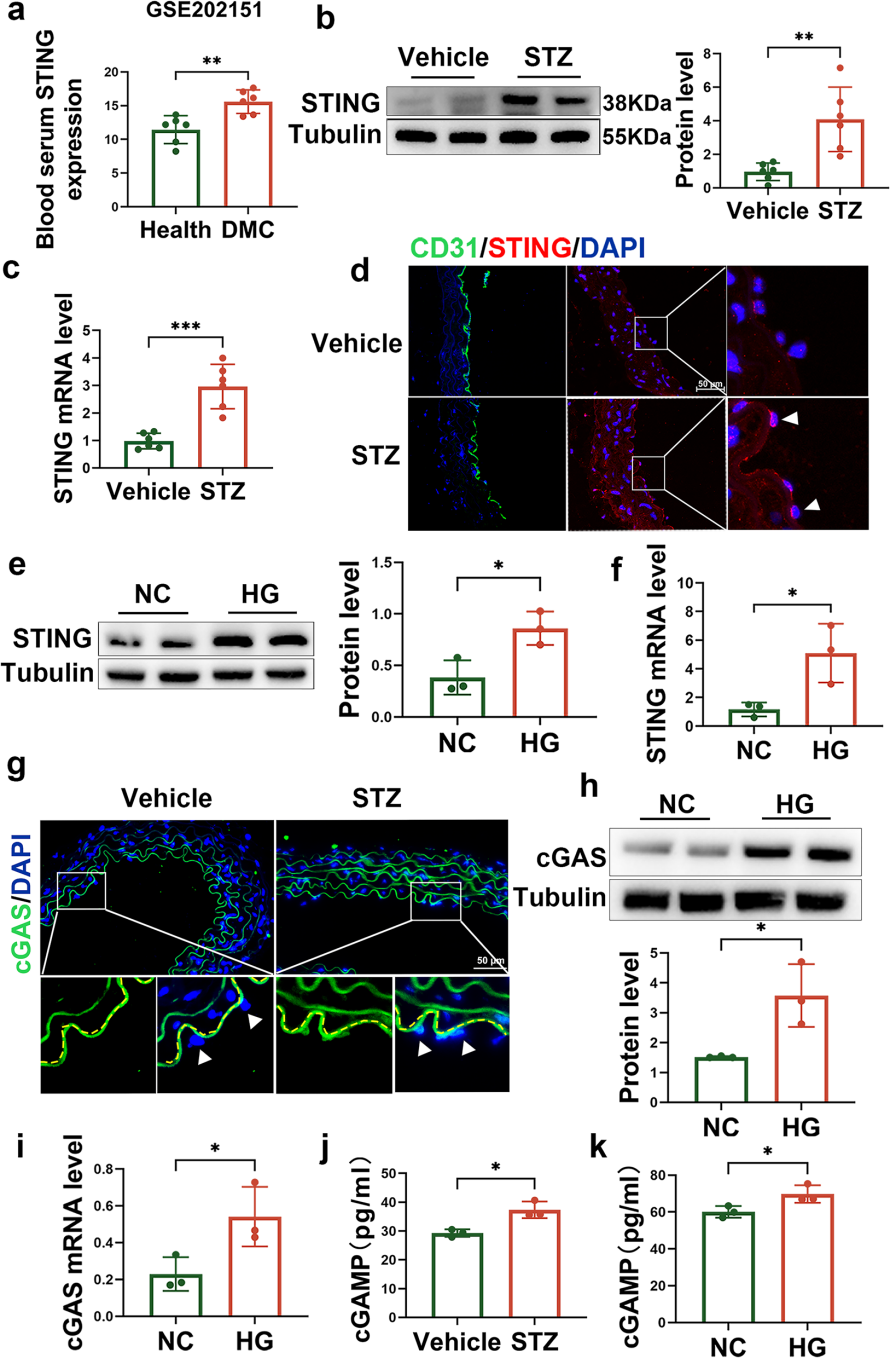
Expression of STING in the diabetic vasculature and high glucose-treated RAECs. **a** The mRNA levels of STING in the leukocytes from healthy controls (*n* = 6) and patients diagnosed with type 2 diabetes with macrovascular complications (DMCs; *n* = 6) based on the GSE160016 dataset. **b** Western blot analysis of STING protein levels in the aortas of vehicle- or STZ-injected mice. **c** qRT–PCR showing STING mRNA levels in the aortas of vehicle- and STZ-injected mice. **d** Representative images showing immunofluorescence staining of CD31 (green) and STING (red) in the aortas of vehicle- and STZ-injected mice. Bars: 50 μm. **e** Western blot analysis of STING protein levels in RAECs treated with HG (40 mmol/L). **f** qRT–PCR analysis showed the mRNA levels of STING in RAECs treated with HG (40 mmol/L). **g** Representative images of immunofluorescence staining of cGAS (green) in the aortas of vehicle- and STZ-injected mice. Bars: 50 μm. **h** Western blot analysis of cGAS protein levels in RAECs treated with HG (40 mmol/L). **i** qRT–PCR analysis of the mRNA levels of cGAS in RAECs treated with HG (40 mmol/L). **j** cGAMP levels were increased in the aortic endothelium of diabetic mice. **k** cGAMP levels were increased in HG-treated RAECs. The results are representative of three independent experiments. The data are presented as the mean ± SD. ∗*P* < 0.05; ∗∗*P* < 0.01; ∗∗∗*P* < 0.001

To investigate whether cGAS-STING is involved in the pathogenesis of diabetic vascular disease, we first searched the available databases and found that STING was significantly upregulated in the leukocytes from type 2 diabetes patients with macrovascular complications (DMCs) in published datasets (Fig. 1a). We then established a diabetic mouse model with high-fat diet (HFD) feeding and streptozotocin (STZ) injection and examined the expression of STING in the aortas of normal and diabetic mice. As shown in Fig. 1b and c, the protein and mRNA levels of STING in the aortas of diabetic mice were significantly increased. Notably, immunofluorescence staining showed that the increase in STING expression was observed only in the aortic endothelial cells of diabetic mice (Fig. 1d). Since hyperglycaemia is the main driver of pathological injury in diabetic complications [3, 20], we next established a glucotoxicity cell model in RAECs treated with HG and examined the effect of high glucose on STING expression. Consistent with the in vivo results, the protein and mRNA levels of STING were significantly increased in aortic endothelial cells treated with HG (Fig. 1e-f). Moreover, cGAS, which is the upstream regulator of STING and is responsible for sensing DNA, was also increased in the aortic endothelium of diabetic mice (Fig. 1g) and HG-treated RAECs (Fig. 1h-i). As expected, cGAMP, which is the mediator between cGAS and STING, was increased in the aortic endothelial cells of diabetic mice and HG-treated RAECs (Fig. 1j-k). These data suggest that hyperglycaemia induces cGAS-STING activation in the aortic endothelial cells of diabetic mice.

### Suppressing STING ameliorates aortic endothelial cell injury in diabetic mice

**Fig. 2:**
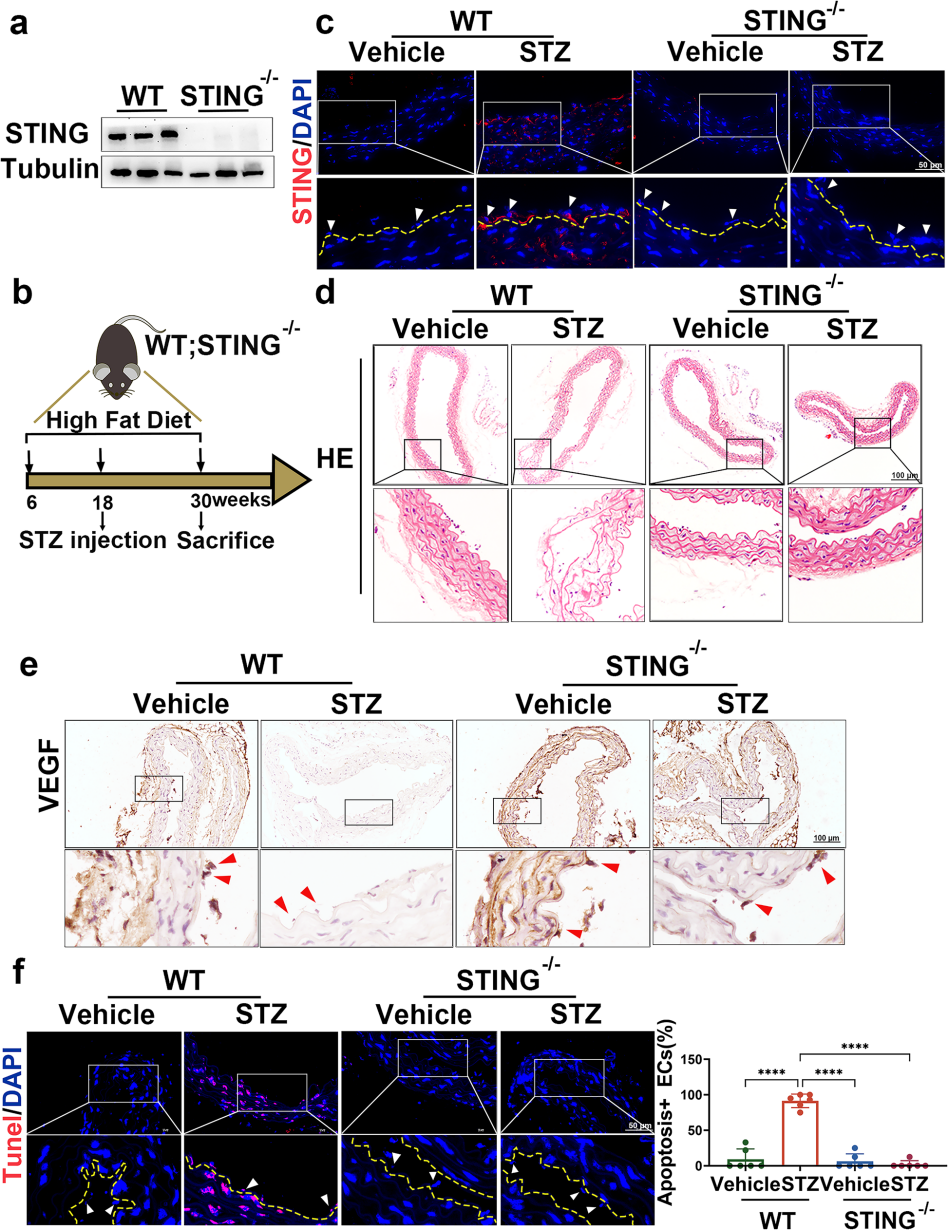
Suppressing STING ameliorates aortic endothelial cell injury in diabetes. **a** Representative Western blot analysis of STING expression levels in the aortas of WT and STING^−/−^ mice. **b** Schematic representation of the diabetic mouse model induced by a high-fat diet combined with STZ. **c** Representative immunofluorescence staining of STING in the aortic endothelium of the indicated mice. The yellow dashed line depicts the aorta endothelium, and the arrow indicates endothelial cells. Bars: 50 μm. Representative images of aortic sections stained with H&E **d**, VEGF **e** and TUNEL **f**. The yellow dotted line indicates the vascular endodermis, and the arrow points to endothelial cells. Bars: 50 μm. The results are representative of three independent experiments. The data are presented as the mean ± SD. ∗*P* < 0.05; ∗∗*P* < 0.01; ∗∗∗*P* < 0.001

To investigate whether endothelial STING activation contributes to diabetes-induced endothelial cell injury, we used STING-knockout (STING^−/−^) mice to establish a diabetic mouse model (Fig. 2a-b Fig. S1a-b). Immunofluorescence staining showed that STING was upregulated in the aortic endothelial cells of diabetic WT mice injected with STZ, and no STING signals were observed in diabetic STING^−/−^ mice injected with either vehicle or STZ (Fig. 2c). Histological analysis showed that the aortic vessels of diabetic WT mice were structurally disrupted and contained broken fibres and fewer endothelial cells, and this effect was reversed in diabetic STING^−/−^ mice (Fig. 2d). To further assess the protective effect of STING knockout on diabetes-induced endothelial cell dysfunction and injury, we examined the endothelial cell-related protein vascular endothelial growth factor (VEGF) and apoptosis levels. As shown in Fig. 2e, VEGF was markedly reduced in the aortic endothelial cells of diabetic WT mice, and this effect was reversed in diabetic STING^−/−^mice. Similarly, TUNEL staining showed that STZ injection increased apoptosis in the aortic endothelial cells of WT mice, whereas in STING^−/−^mice, no increase in apoptosis was induced by STZ injection compared with vehicle injection (Fig. 2f). Taken together, these results indicate that suppressing STING ameliorates aortic endothelial cell injury in diabetic mice.

### STING activation mediates high glucose-induced aortic endothelial cell dysfunction in vitro

**Fig. 3:**
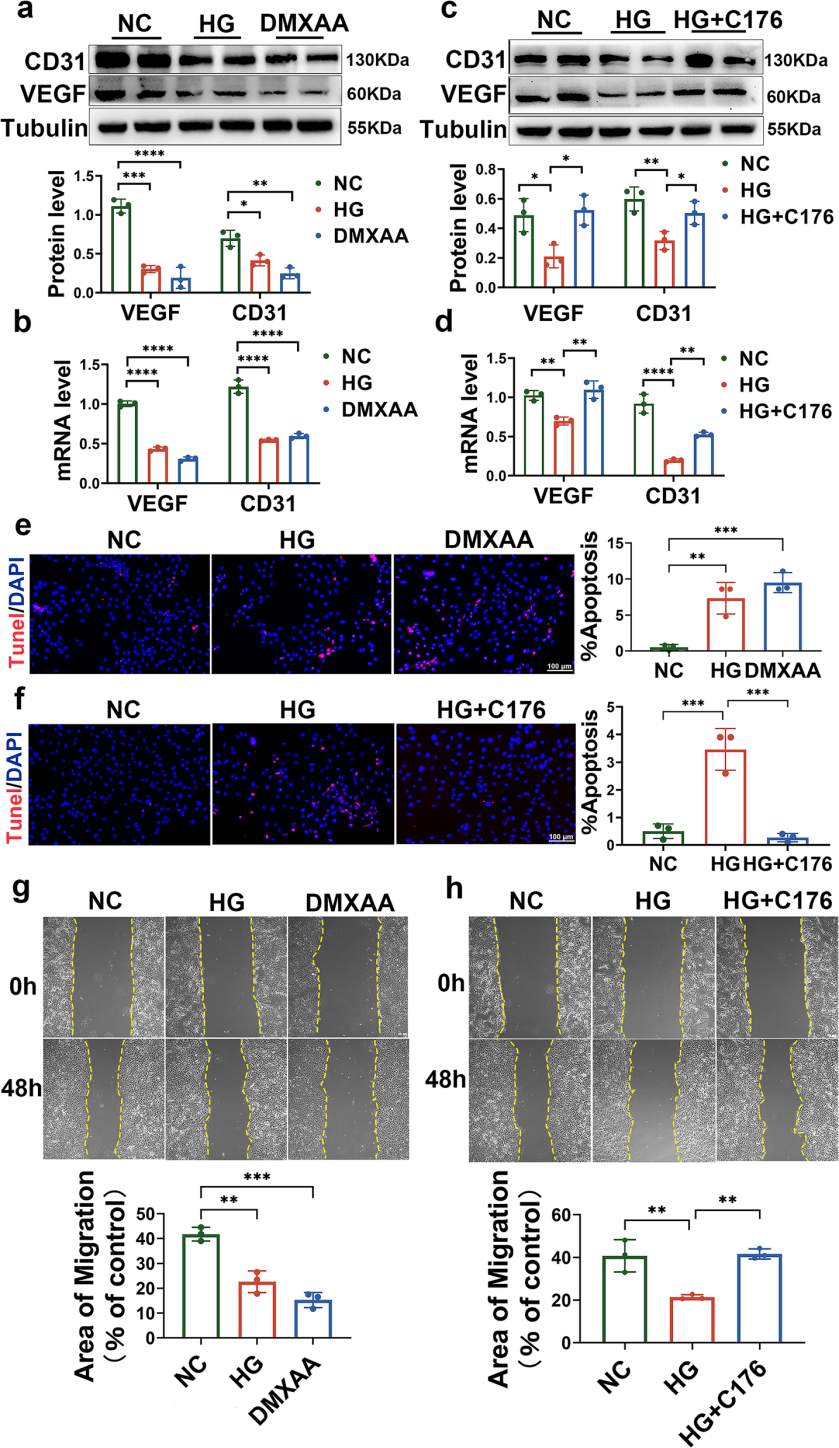
STING mediates high glucose-induced aortic endothelial cell dysfunction in vitro*.* **a** Representative Western blot analysis of VEGF and CD31 in RAECs treated with HG (40 mmol/L) and DMXAA (50 μg/ml). **b** qRT–PCR analysis of the expression of VEGF and CD31 in RAECs treated with HG and DMXAA. **c**-**d** RAECs were treated with HG in the absence or presence of C176 (5 nmol/L) for 72 h. Western blotting **c** and qRT–PCR **d** were performed to detect VEGF and CD31. **e** TUNEL staining showing the apoptotic levels of RAECs treated with HG and DMXAA. Bars: 100 μm. **f** TUNEL staining showing the apoptotic levels of HG-treated RAECs in the absence or presence of C176. Bars: 100 μm. **g** Representative images and quantification of migration of RAECs treated with HG and DMXAA. Bars: 50 μm. **h** Representative images and quantification of the migration of RAECs stimulated by HG in the absence or presence of C176. Bars: 50 μm. The results are representative of three independent experiments. The data are presented as the mean ± SD. ∗*P* < 0.05; ∗∗*P* < 0.01; ∗∗∗*P* < 0.001

Given that high glucose induced STING activation (Fig. 1e-f) in aortic endothelial cells, we next investigated whether STING activation mediated glucotoxicity-induced aortic endothelial cell injury. To this end, we used the STING inhibitor C-176 and the STING activator DMXAA to mimic the gain- or loss-of-function of STING, respectively. Consistent with the effects of HG, DMXAA treatment reduced the expression levels of the endothelial cell markers CD31 and VEGF in RAECs (Fig. 3a-b). In contrast, HG combined with C-176 reversed the reduction in CD31 and VEGF in RAECs treated with HG alone (Fig. 3c-d), suggesting that HG-induced STING activation could contribute to HG-induced endothelial cell dysfunction. In addition, we examined the role of STING in endothelial cell migration and apoptosis. The wound healing assay showed that DMXAA alone significantly inhibited endothelial cell migration, similar to HG treatment (Fig. 3e). In contrast, HG treatment inhibited the migration of endothelial cells, and this effect could be reversed by C-176 treatment (Fig. 3f). Moreover, the TUNEL assay showed that DMXAA alone significantly promoted endothelial cell apoptosis, similar to HG treatment (Fig. 3g), and HG plus C-176 reversed HG-induced apoptosis (Fig. 3h). These results suggest that STING activation mediates high glucose-induced aortic endothelial cell dysfunction in vitro*.*

### Suppressing STING inhibits diabetes-induced activation of the IRF3/NF-κB pathway in aortic endothelial cells

**Fig. 4:**
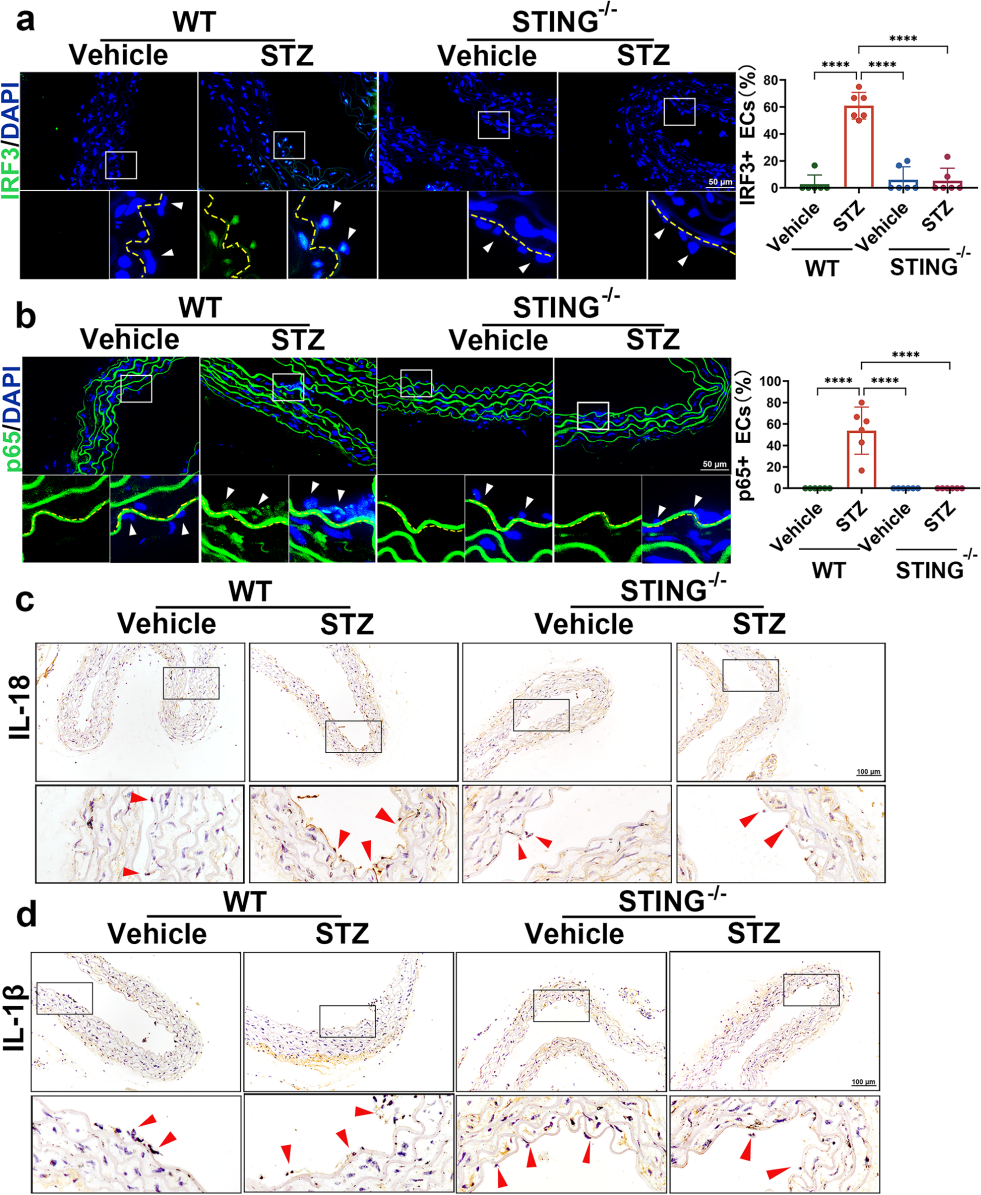
Suppressing STING inhibits diabetes-induced inflammatory activation in aortic endothelial cells. **a**-**b** Representative immunofluorescence images and quantification of IRF3 **a** and p65 **b** in the aortic endothelium of mice in the indicated groups. The yellow dotted line indicates the aortic endothelium, and the arrow indicates endothelial cells. **c**-**d** Representative immunohistochemical staining of IL-18 and IL-1β in the aortas of mice in the indicated groups. The arrows indicate endothelial cells. The results are representative of three independent experiments. The data are presented as the mean ± SD. ∗*P* < 0.05; ∗∗*P* < 0.01; ∗∗∗*P* < 0.001

Given that hyperglycaemia-induced chronic inflammation is one of the drivers of diabetic complications [3, 20] and STING has been reported to mediate innate host immunity through downstream inflammatory pathways such as IRF3/NF-κB signalling [17], we hypothesized that STING activation could mediate diabetes-induced aortic endothelial cell injury via the IRF3/NF-κB inflammatory signalling pathway. To test this hypothesis, we examined the activity of the IRF3/NF-κB pathway in WT or STING^−/−^ mice injected with vehicle or STZ. As expected, in diabetic WT mice, IRF3 and p65 levels were increased in the nuclei of aortic endothelial cells, where these factors function as transcriptional activators of inflammatory factors. Notably, the diabetes-induced increase in nuclear IRF3 and p65 was completely blocked by STING suppression in diabetic STING^−/−^ mice (Fig. 4a-b, Fig. S2a). cGAS, as the upstream of STING, was significantly activated in endothelial cells of diabetic mice, while knockout of STING had no effect on the expression of cGAS in endothelial cells of diabetic mice (Fig. S2b). Consistently, immunohistochemical staining showed that the expression of IL-1β and IL-18, which are the downstream targets of IRF3 and p65, was increased in the aortic endothelial cells of diabetic WT mice, and this effect was reversed by STING suppression in diabetic STING^−/−^ mice (Fig. 4c-d). Taken together, these results indicate that the suppression of STING ameliorates diabetes-induced aortic endothelial cell injury through IRF3/NF-κB-mediated inflammatory activation.

### Suppressing STING inhibits high glucose-induced activation of the IRF3/NF-κB pathway in vitro

**Fig. 5:**
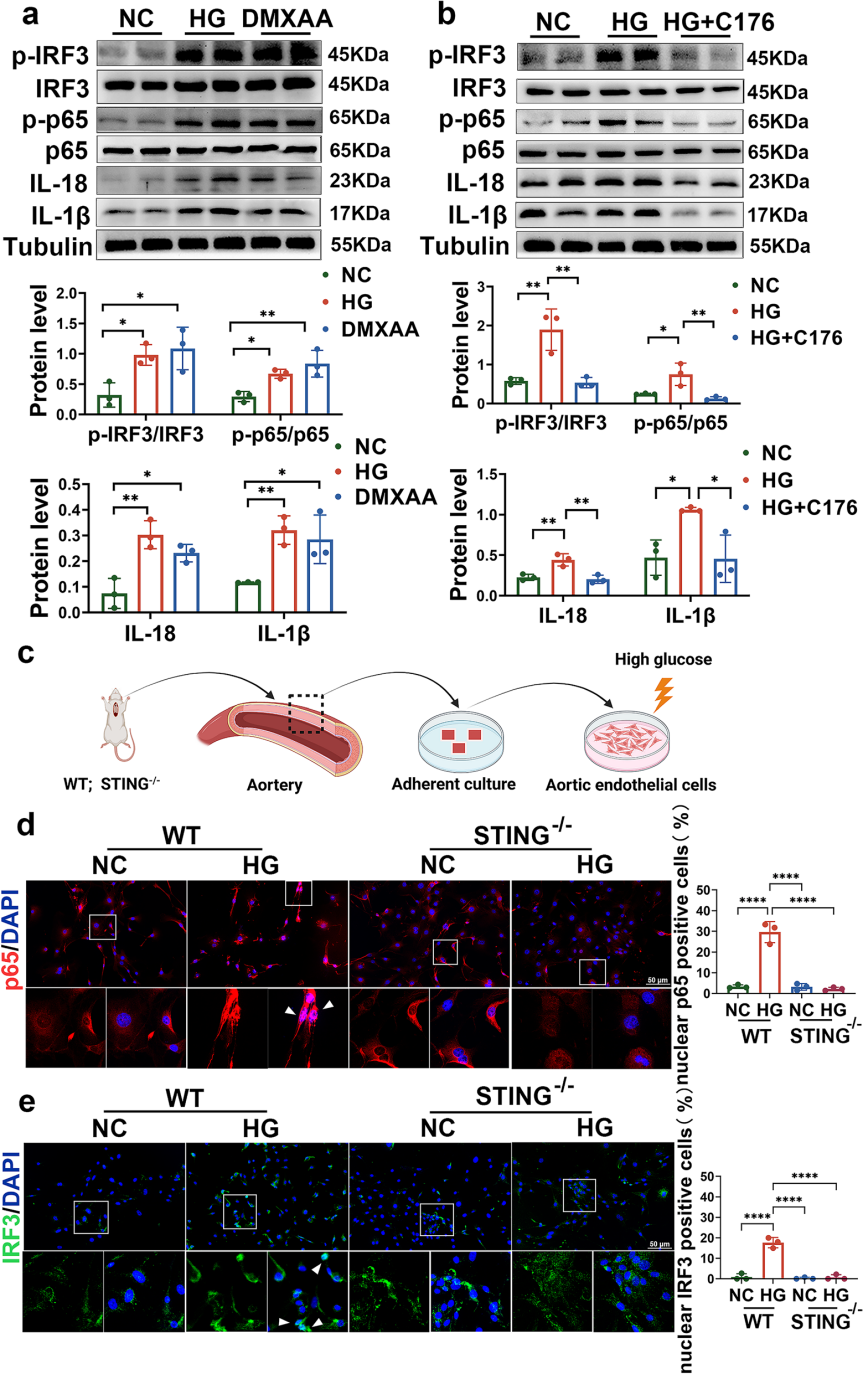
Suppressing STING inhibits HG-induced inflammatory activation in vitro*.* **a** Representative Western blot analysis and density quantification of phosphorylated IRF3, total IRF3, phosphorylated p65, total p65, IL-1β, and IL-18 in RAECs treated with HG and DMXAA. **b** RAECs were treated with HG in the absence or presence of C176 for 72 h. Western blot analysis and density quantification were performed to detect phosphorylated IRF3, total IRF3, phosphorylated p65, total p65, IL-1β and IL-18 in the indicated groups. **c** Schematic representation of primary aortic endothelial cell extraction. **d**-**e** Immunofluorescence staining showing the location of IRF3 and p65 in HG-treated primary aortic endothelial cells from WT and STING^−/−^ mice. Bars: 50 μm. The results are representative of three independent experiments. The data are presented as the mean ± SD. ∗*P* < 0.05; ∗∗*P* < 0.01; ∗∗∗*P* < 0.001

Next, we examined whether STING activation mediates glucotoxicity through the IRF3/NF-κB signalling pathway in HG-treated aortic endothelial cells, as we observed in vivo. As shown in Fig. 5a, DMXAA treatment increased the phosphorylation levels of IRF3 and NF-κB, which help these factors enter the nucleus to act as transcriptional regulators, which is consistent with what we observed in HG-treated aortic endothelial cells (Fig. 5a). In addition, their downstream targets IL-18 and IL-1β were elevated in DMXAA-treated aortic endothelial cells, as well as those treated with HG (Fig. 5a). In contrast, HG plus C-176 reversed the increase in phosphorylated IRF3 and NF-κB, as well as the expression of IL-18 and IL-1β in HG-treated RAECs (Fig. 5b). Similarly, we found that HG can also activate the STING/IRF3/NF-κB pathway in human aortic endothelial cells (HAECs), and C176 can inhibit the activation of the pathway caused by HG (Fig. S3a-c). In addition to C-176-mediated STING inhibition, we also isolated primary endothelial cells from the aortic intima of WT and STING^−/−^ mice and subjected them to HG treatment in vitro (Fig. 5c)*.* Immunofluorescence staining showed that IRF3 and p65 levels were increased in the nuclei of HG-treated WT endothelial cells, and these effects were completely reversed by STING suppression in HG-treated STING^−/−^ endothelial cells (Fig. 5d-e). Collectively, these results demonstrate that STING activation mediates high glucose-induced aortic endothelial cell injury through IRF3/NF-κB-mediated inflammation.

### HG-induced mtDNA release activates the STING signalling pathway in aortic endothelial cells

**Fig. 6:**
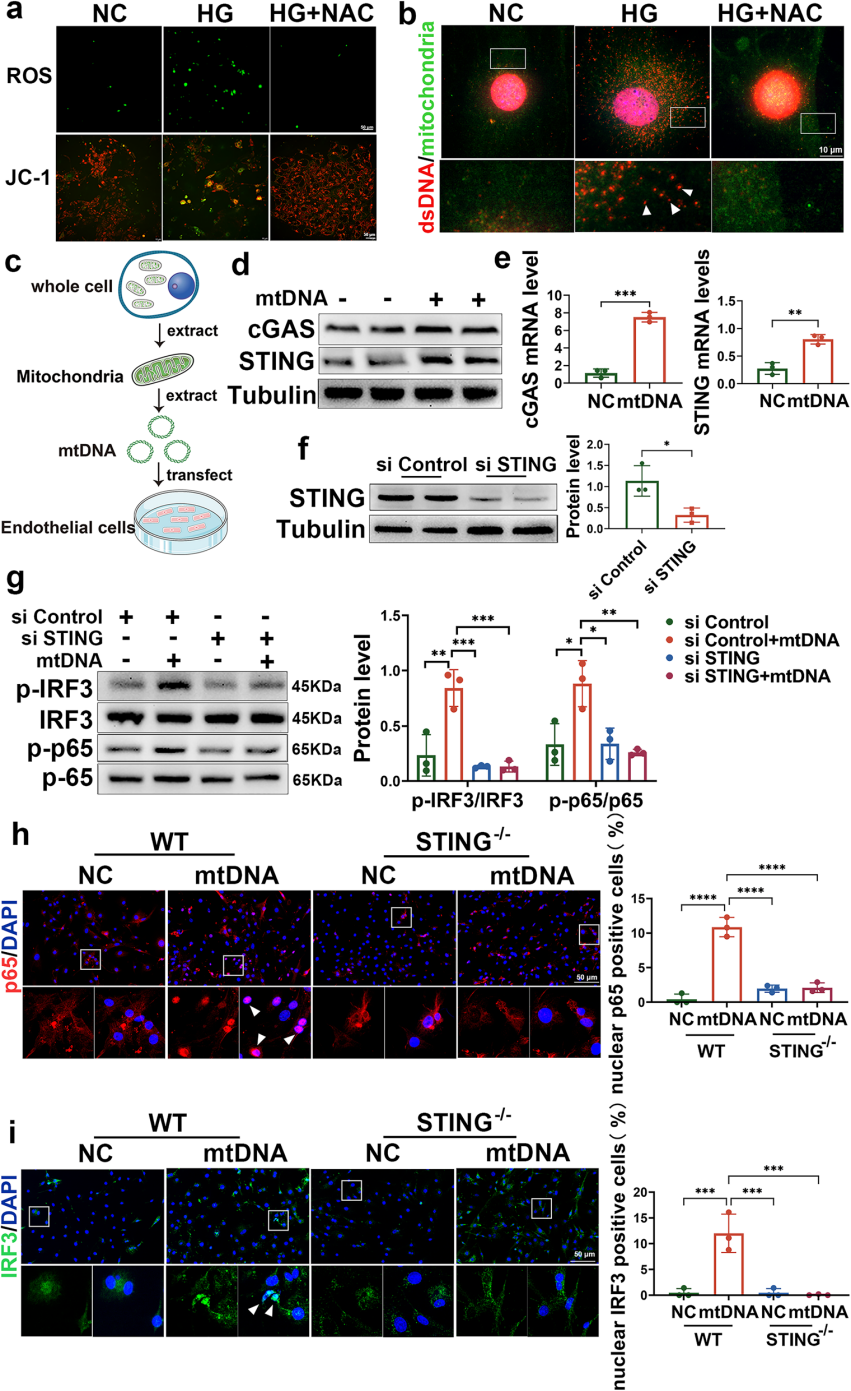
HG-induced mtDNA release activates the STING signalling pathway in aortic endothelial cells. **a** The DCFH-DA probe was used to measure ROS levels in HG-induced primary aortic endothelial cells with or without NAC treatment (5 mmol/l) for 2 h. Bars: 50 μm. JC-1 staining revealed the mitochondrial membrane potential of HG-induced primary aortic endothelial cells with or without NAC treatment. Bars: 50 μm. **b** A coimmunostaining assay was used to detect the location of dsDNA (red) and mitofilin (green) in the cytoplasm of primary endothelial cells stimulated by high glucose with or without NAC treatment. Bars: 10 μm. **c** Flow chart of mtDNA extraction and transfection. **d**-**e** Western blotting and qRT–PCR analysis of the protein and mRNA levels of cGAS and STING in RAECs 24 h after transfection with 3 μg of mtDNA. **f** Representative Western blot showing STING in RAECs transfected with control siRNA or STING siRNA. **g** Western blot and density quantification showing the protein levels of phosphorylated IRF3, total IRF3, phosphorylated p65 and total p65 in RAECs transfected with control or STING siRNA in the absence or presence of mtDNA. **h**-**i** Immunofluorescence staining showing the location of IRF3 and p65 in mtDNA-treated primary aortic endothelial cells from WT and STING−/− mice. Bars: 50 μm. The results are representative of three independent experiments. The data are presented as the mean ± SD. ∗*P* < 0.05; ∗∗*P* < 0.01; ∗∗∗*P* < 0.001

As a DNA sensor, the cGAS-STING system mediates innate host immunity by sensing exogenous DNA and metabolic diseases by sensing endogenous DNA [21]. Our previous study revealed that lipotoxicity-induced mtDNA release activated the cGAS-STING pathway in cardiomyocytes in obesity-related diabetes [14]. Therefore, we examined whether glucotoxicity-induced mtDNA release activated the STING signalling pathway in aortic endothelial cells. We first isolated primary endothelial cells and treated them with HG. As previously reported, HG treatment induced ROS overproduction and decreased mitochondrial membrane potential in aortic endothelial cells, as determined by JC-1 staining, and these effects could be reversed by the ROS inhibitor NAC (Fig. 6a). Coimmunostaining of double-stranded DNA (dsDNA) and mitochondrial markers showed that HG treatment increased cytosolic mtDNA, and this effect was reversed by the ROS inhibitor NAC (Fig. 6b). These data indicate that HG treatment leads to mtDNA release through ROS overproduction. Next, we purified mtDNA from the cell lysate and added it to the culture medium of RAECs (Fig. 6c, Fig. S4a). As shown in Fig. 6D, mtDNA treatment significantly induced the expression of cGAS, which is responsible for DNA sensing in the cGAS-STING system, and STING (Fig. 6d-e). To determine whether mtDNA activates STING signalling in aortic endothelial cells, we first established STING knockdown cells via STING siRNA transfection (Fig. 6f) and treated them with purified mtDNA or vehicle. Notably, mtDNA treatment activated downstream STING signalling and increased the phosphorylation of IRF3 and p65 in RAECs transfected with control siRNA; this effect was abolished in endothelial cells transfected with STING siRNA (Fig. 6g). Similarly, mtDNA activates STING in HAECs, and the downstream IRF3/NF-κB is also activated. However, this effect is reversed in HAECs transfected with STING siRNA (Fig. S4b-d). In addition, mtDNA treatment activated downstream STING signalling and increased the nuclear localization of IRF3 and p65 in primary aortic endothelial cells from WT mice; this effect was abolished in cells from STING^−/−^ mice (Fig. 6h). These findings suggest that HG-induced mtDNA release activates STING and its downstream signalling under diabetic conditions.

## Discussion

**Fig. 7:**
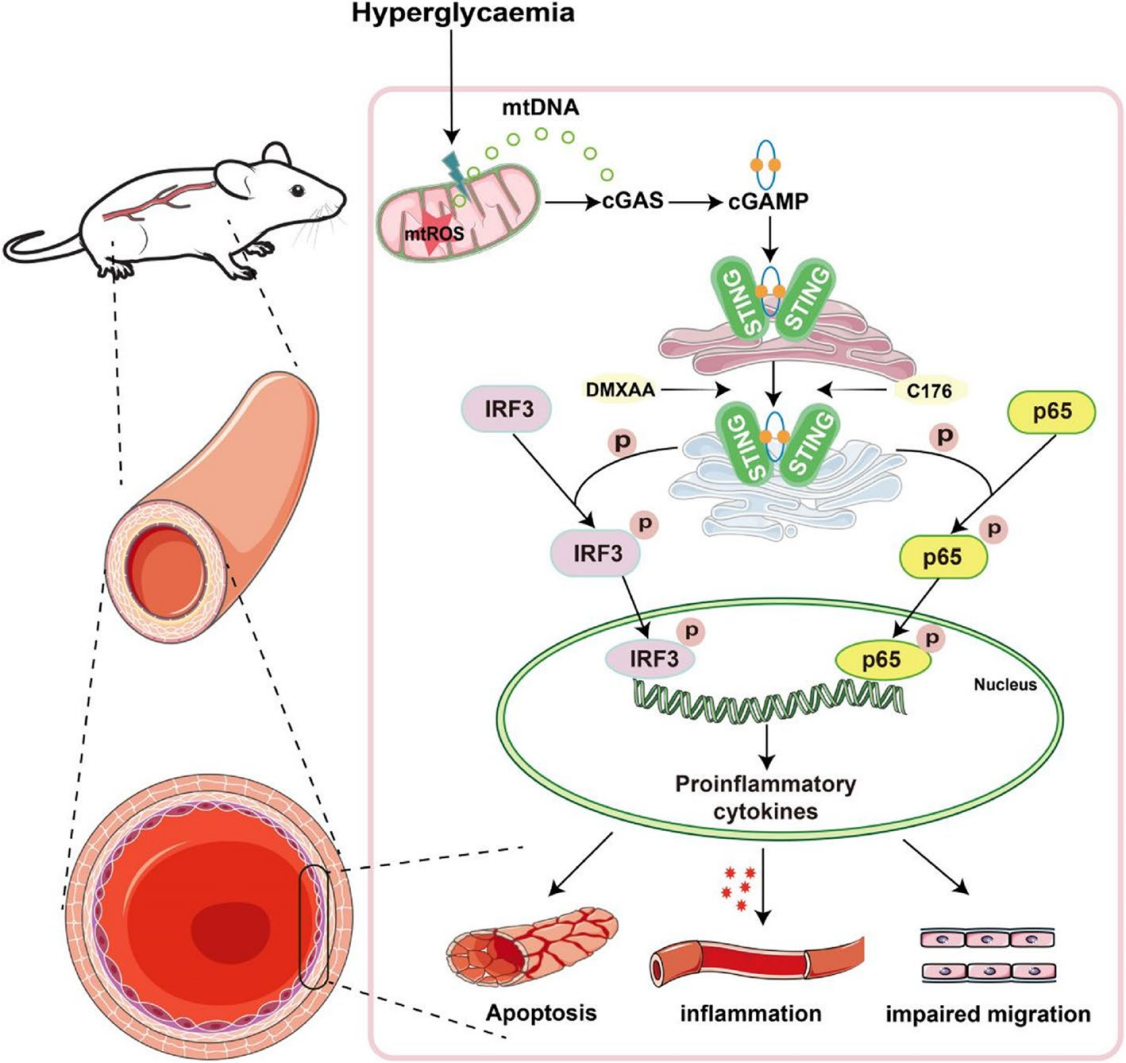
Schematic illustration of the mechanism by which hyperglycaemia-induced activation of the cGAS-STING signalling pathway promotes aortic endothelial cell injury in diabetic macrovascular complications. Hyperglycaemia-induced mtROS production can cause mtDNA leakage into the cytoplasm, which is recognized by cGAS and subsequently activates STING by shifting from the endoplasmic reticulum to the Golgi apparatus. Activated STING promotes the phosphorylation and nuclear transfer of IRF3 and p65, which ultimately leads to inflammatory reactions and apoptosis in aortic endothelial cells in the context of diabetes

STING has been reported to play important roles in innate immune and metabolic diseases, but the association between STING and diabetic vascular disease is unknown. Our study reveals a previously unrecognized mechanism of diabetic vascular endothelial injury associated with STING signalling. We first showed that STING was specifically increased in aortic endothelial cells under diabetic conditions. Second, suppressing STING alleviated hyperglycaemia-induced aortic endothelial cell injury in vivo and in vitro*.* Finally, cGAS-STING signalling was activated by hyperglycaemia-induced mtDNA release, which promoted the expression of inflammatory cytokines through the IRF3/NF-kB pathway and ultimately resulted in diabetic aortic endothelial cell dysfunction (Fig. 7). Collectively, these data strongly suggest that STING is a potential target for the treatment of diabetic vascular disease.

Despite being a DNA sensing-related protein that was originally identified in the innate immune system, STING has also been widely reported to be involved in various vascular diseases. In a hypercholesterolaemia mouse model, STING was shown to be increased in macrophages in the aorta. Genetic deletion or pharmacological blockade of STING ameliorated atherogenesis in Apoe^−/−^ mice [16]. In another atherosclerosis model induced by chronic kidney injury, STING activation in vascular SMCs triggers the IFN-I response, thereby promoting the pathogenesis of CKD-associated plaque vulnerability [22]. Moreover, significant activation of the STING pathway was observed in SMCs and macrophages in aortic tissue from humans with AAD, and STING activation-induced SMC death triggers the STING pathway in macrophages [15]. Unlike previous studies, we observed that STING expression was only increased in aortic endothelial cells but not SMCs or macrophages in diabetes, as was found in AS or AAD (Fig. 1). This suggests selective activation of STING in different cell types under different pathological conditions associated with vascular disease. Consistent with previous studies, we also found that suppressing STING protected against hyperglycaemia-induced endothelial cell injury, further indicating the therapeutic potential of STING in vascular disease.

As a gene that is highly expressed in endothelial cells in biopsy samples of lesioned skin from patients with STING-associated vasculopathy with onset in infancy (SAVI) and in an endothelial cell lines [23], STING has been reported to be activated in various endothelial cells under different pathological conditions. In endothelial cells from human umbilical veins, cGAMP stimulation leads to endothelial dysfunction and apoptosis [23]. Moreover, in endothelial cells from the lung, STING activation with the STING agonist 10-carboxymethyl-9-acridanone (CMA) impaired endothelial proliferation after LPS challenge [17], which was consistent with the observation that STING activation by DMXAA induced apoptosis in RAECs in our study. In addition, hyperlipidaemia has also been reported to activate the STING pathway in endothelial cells in metabolic diseases. In retinal endothelial cells in diabetic retinopathy, hyperlipidaemia induces proinflammatory responses by activating the STING pathway via IRE1α-XBP1 [24]. Free fatty acids trigger endothelial inflammation through the STING–IRF3 axis in adipose tissue in diet-induced obesity in vivo [18] and inhibit endothelial angiogenesis through cGAS-STING-IRF3 signalling in vitro [25]. However, the role of the STING pathway in hyperglycaemia-induced endothelial cell dysfunction in diabetes has not been reported. In our study, we revealed that high glucose-induced cytosolic mtDNA release activated cGAS-STING signalling and the downstream inflammatory pathway in aortic endothelial cells. Furthermore, to exclude the interference of high fatty acid levels in our mouse model, we established a glucotoxicity cell model and found that suppressing STING with specific inhibitors or microRNAs markedly reversed high glucose-induced endothelial cell injury, indicating that in addition to high fatty acids, high glucose could also trigger the STING pathway in endothelial cells.

Although diabetic vascular disease is one of the major causes of death in diabetic patients, there is a lack of mechanism-based therapeutic drugs because intensive glycaemic control does not eradicate vascular injury [26]. In this study, we identified STING as a potential target and used C-176, which is a highly efficient and selective inhibitor [27], to block STING activation in HG-treated RAECs. Our results showed that C-176 could inhibit HG-induced STING expression, as well as downstream IRF3/NF-κB signalling, which is consistent with previous studies on the effect of C-176 on renal and cardiovascular disease [9, 14]. These data suggest that STING inhibition is a non-hypoglycaemic therapeutic strategy in the treatment of diabetic artery injury. Notably, DMXAA is a STING agonist that can be used in tumour treatment to destroy tumour vasculature by stimulating cytokine and chemokine production in tumour-related macrophages and endothelial cells [28, 29]. Additionally, DMXAA induces endothelial cell apoptosis, which increases vascular endothelial permeability and reduces tumour blood flow [30]. Consistently, we found that DMXAA treatment activated the STING/IRF3/NF-κB pathway and induced apoptosis in RAECs, similar to HG treatment. Therefore, due to the lack of specific tissue targeting, most STING agonists not only activate STING in blood vessels but also cause damage to blood vessels. Recent studies have shown that nanoparticles can accurately deliver STING agonists to tumour blood vessels, which leads to local and specific antitumour immune responses [31, 32]. In summary, STING inhibition is a promising strategy for the treatment of diabetic vascular disease, but care should be taken when STING activators such as DMXAA are used for tumour therapy.

In conclusion, we examined the role of STING in the pathogenesis of diabetic vascular disease in vivo and the glucotoxicity of RAECs in vitro*.* We observed that STING expression was increased specifically in the endothelial cells of diabetic arteries, as well as in HG-treated RAECs. Genetic deletion of STING ameliorated diabetes-induced aortic endothelial injury, and pharmacological inhibition of STING reversed HG-induced migration dysfunction and apoptosis in RAECs. Furthermore, we revealed that the hyperglycaemia-induced cytosolic translocation of mtDNA leads to cGAS-STING-dependent IRF3/NF-kB activation, resulting in inflammation and apoptosis. We concluded that mtDNA release-induced STING activation mediates hyperglycaemia-induced aortic endothelial cell injury and identified STING as a potential target in the treatment of diabetic vascular disease.

### Supplementary information

**Additional file 1.**

## Data Availability

The data that support the findings of this study are available from the corresponding author upon reasonable request.
