# Supplementary material for: Hyperglycemia-induced STING signaling activation leads to aortic endothelial injury in diabetes
Source: Cell Commun Signal. 2023 Dec 21;21:365. doi: 10.1186/s12964-023-01393-w (PMC10734150; doi:10.1186/s12964-023-01393-w)
Supplement: Supplementary file 2 — Additional file 1. [file 12964_2023_1393_MOESM1_ESM.docx]

**Supplementary Fig. 1**

**
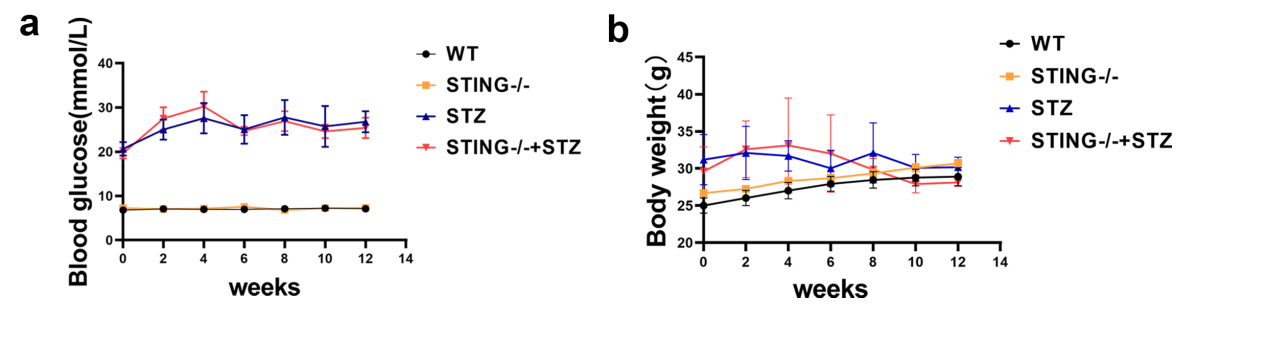
**

(a-b) Changes of blood glucose (a) and body weight (b) in WT and STING^-/-^ mice after STZ injection.

**Supplementary Fig. 2**


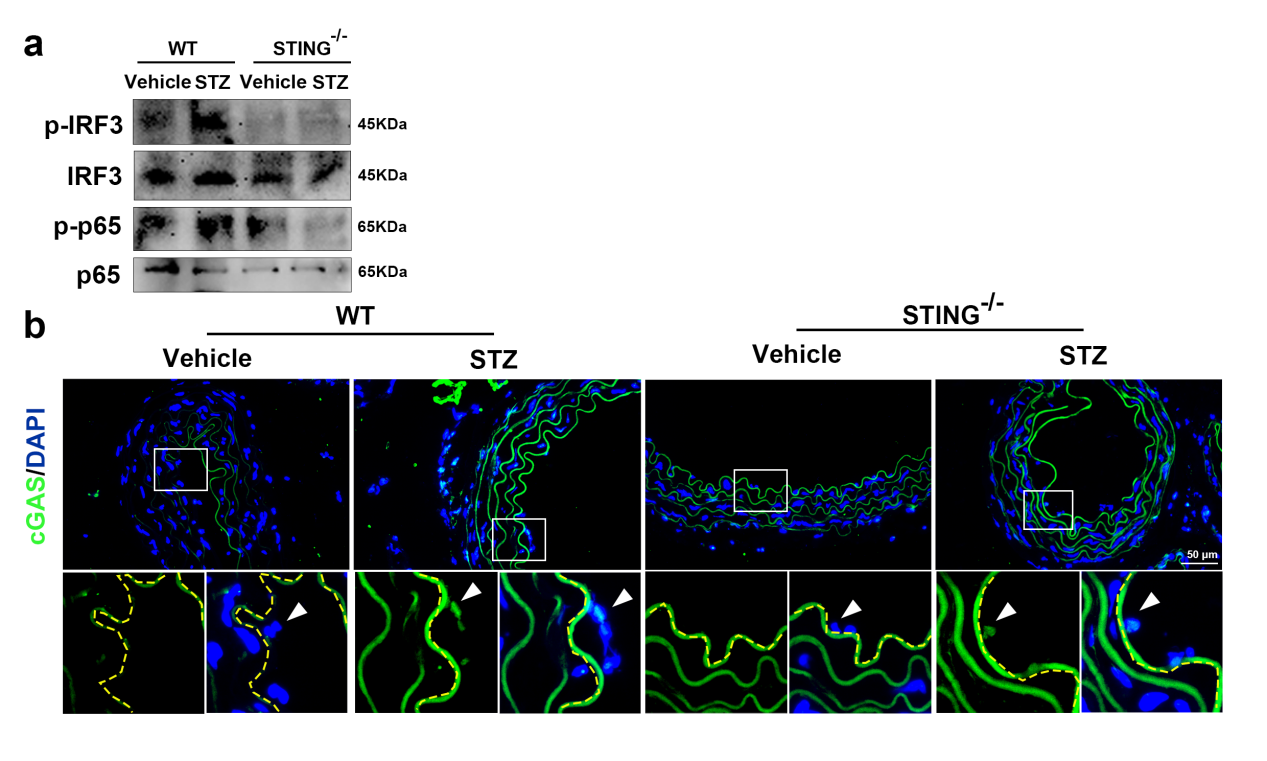


1. Western blot analysis of STING protein levels in the aortas of WT or STING^-/-^ mice after STZ injection. (b) Representative immunofluorescence staining of cGAS in the aortic endothelium of the indicated mice. The yellow dashed line depicts the aorta endothelium, and the arrow indicates endothelial cells. Bars: 50 μm.

**Supplementary Fig. 3**


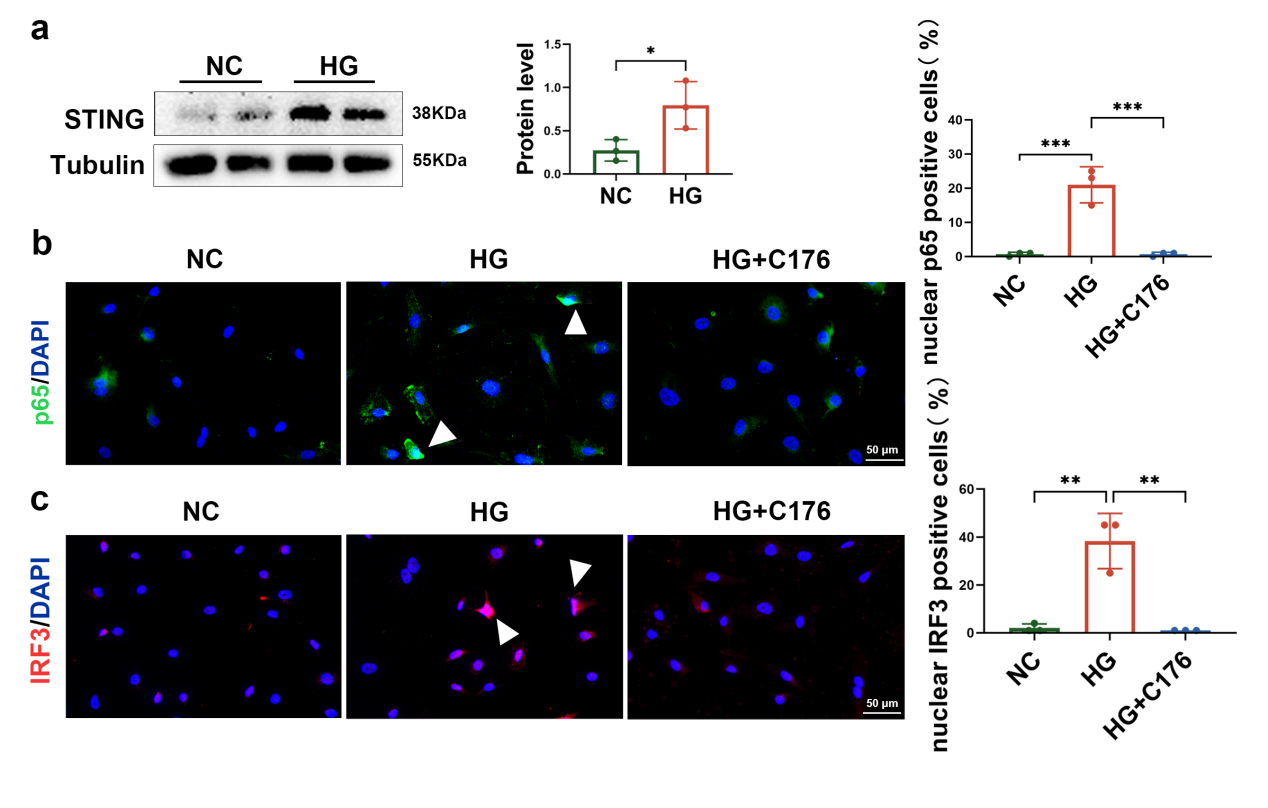


(a)Western blotting analysis for the STING protein levels in human aortic endothelial cells (HAECs) treated with HG (40mmol/L). (b) Representative images of immunofluorescence staining of p65 (green) in the HG-treated HAECs in the absence or presence of C176. Bars: 50 μm. (c) Representative images of immunofluorescence staining of IRF3 (red) in the HG-treated HAECs in the absence or presence of C176. Bars: 50 μm. All results are representative of three independent experiments. Date are presented as mean ±SD. ∗P<0.05; ∗∗P <0.01; ∗∗∗P <0.001.

**Supplementary Fig. 4**

**
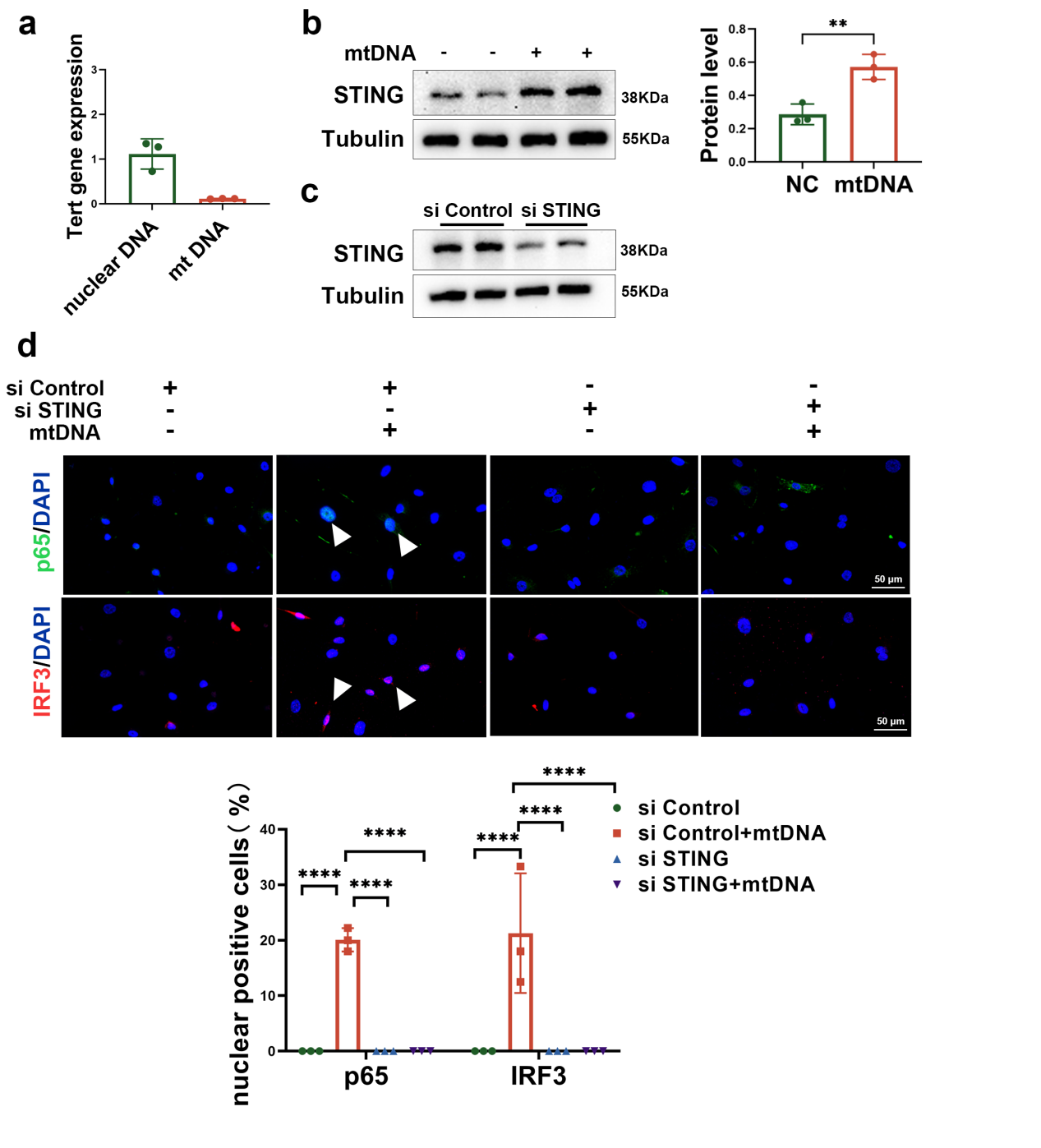
**

1. Quantitative analysis of nuclear gene Tert expression in nuclear DNA and mtDNA. (b) Western blotting showed the protein levels of STING in HAECs 24 h after transfection with 3 μg mtDNA. (c) Representative western blot for STING in HAECs transfected with control siRNA or STING siRNA.Bars: 50 μm. (d) Immunofluorescence staining showed the location of IRF3 and p65 in HAECs transfected with control or STING siRNA in absence or presence of mtDNA.Bars: 50 μm. All results are representative of three independent experiments. Date are presented as mean ±SD. ∗P<0.05; ∗∗P <0.01; ∗∗∗P <0.001.
